# Efficient Planar Perovskite Solar Cells Using Passivated Tin Oxide as an Electron Transport Layer

**DOI:** 10.1002/advs.201800130

**Published:** 2018-03-25

**Authors:** Yonghui Lee, Seunghwan Lee, Gabseok Seo, Sanghyun Paek, Kyung Taek Cho, Aron J. Huckaba, Marco Calizzi, Dong‐won Choi, Jin‐Seong Park, Dongwook Lee, Hyo Joong Lee, Abdullah M. Asiri, Mohammad Khaja Nazeeruddin

**Affiliations:** ^1^ Group for Molecular Engineering of Functional Materials Ecole Polytechnique Fédérale de Lausanne CH‐1951 Sion Switzerland; ^2^ Division of Materials Science and Engineering Hanyang University 222 Wangsimni‐ro Seongdong‐gu Seoul 133‐791 Korea; ^3^ Laboratory of Materials for Renewable Energy Ecole Polytechnique Fédérale de Lausanne CH‐1951 Sion Switzerland; ^4^ Division of Physics and Applied Physics School of Physical and Mathematical Science Nanyang Technological University Singapore 637371 Singapore; ^5^ Department of Chemistry, and Bioactive Material Sciences Chonbuk National University Jeonju 561‐756 Korea; ^6^ Center of Excellence for Advanced Materials Research (CEAMR) King Abdulaziz University P. O. Box 80203 Jeddah 21589 Saudi Arabia

**Keywords:** atomic layer deposition, passivation, perovskites, planar perovskite solar cells, SnO_2_ electron transporting layers

## Abstract

Planar perovskite solar cells using low‐temperature atomic layer deposition (ALD) of the SnO_2_ electron transporting layer (ETL), with excellent electron extraction and hole‐blocking ability, offer significant advantages compared with high‐temperature deposition methods. The optical, chemical, and electrical properties of the ALD SnO_2_ layer and its influence on the device performance are investigated. It is found that surface passivation of SnO_2_ is essential to reduce charge recombination at the perovskite and ETL interface and show that the fabricated planar perovskite solar cells exhibit high reproducibility, stability, and power conversion efficiency of 20%.

Over the past few years, there have been tremendous research and development in perovskite solar cells.^1^ Such a blistering interest is due to ease in the fabrication of perovskite solar cells using low‐cost solution processes methods, which enabled high power conversion efficiencies (PCE) over 22%.^2^, ^3^, ^4^, ^5^ For perovskite solar cells in an n‐i‐p configuration, TiO_2_ has been widely used as an electron transporting layer (ETL) because of its favorable bandgap edge positions in relation to perovskite conduction band, and reduced processing costs.^6^ However, high‐temperature deposition and poor charge collection at the TiO_2_/perovskite interface are hindering for wider application of TiO_2_ in planar perovskite solar cells.^7^, ^8^, ^9^ In this respect, SnO_2_ that has a higher electron mobility is an excellent candidate to replace TiO_2_.^10^ A wide bandgap ranging from 3.6 to 4.1 eV and chemical stability show a great potential of SnO_2_ as an efficient electrode.^11^ Nevertheless, metal‐like nature shown in degenerated semiconductors seems to generate a serious shunting pathway decreasing the fill factor (FF) and an open‐circuit voltage (*V*_oc_).^12^, ^13^ The recent data on surface treatments in perovskite solar cells signify the importance of surface passivation for the SnO_2_ layer.^13^, ^14^ Within this understanding, it is not surprising to find that the SnO_2_ layer often positions as a sublayer that can function as a surface leveler and assisting ones for the primary ETL such as TiO_2_ or phenyl‐C_60_‐butyric acid methyl ester (PCBM) in normal and inverted type perovskite solar cells.^15^ We have demonstrated that a severe drop of the *V*_oc_ and the PCE in the devices using solution‐processed SnO_2_ films heat‐treated at high temperature.^13^ The low PCE is due to the deteriorated hole‐blocking ability of the SnO_2_ ETL caused by loss of the self‐passivating SnOCl_2_. In this article, we prove that the low‐temperature process for the SnO_2_ ETL is an indispensable choice to reduce charge recombination for photovoltaic applications.

The atomic layer deposition (ALD) technique is an efficient way to prepare SnO_2_ films at low temperature, which is based on self‐limiting surface reactions by exposing sequentially on the substrate with various precursors and reactants. It is enabling precise thickness control at the angstrom or monolayer level and deposition on high aspect ratio nanostructures with excellent step coverage.^16^ Since this technique is known to provide a good film and device performance compared with solution‐processed ones,^17^ we prepared SnO_2_ layers using the ALD method by modulating deposition or postannealing temperature, and showed how temperature can influence on optical, chemical, and electrical properties of the SnO_2_ film along with the device performance.

The microstructures from scanning electron microscope (SEM) of the ALD SnO_2_ films deposited at 120 °C and the complete perovskite device are displayed in **Figure** **1**a. A smooth and conformal SnO_2_ layer without pinholes is observed over the fluorine‐doped SnO_2_ (FTO) top‐surface. The complete cell of a planar structure is composed of FTO/ALD SnO_2_/perovskite/poly triarylamine (PTAA)/gold layers. In this structure, ALD SnO_2_ functions as an ETL, while perovskite, PTAA, and gold work as a light absorber, a hole transporting material (HTM), and a counter electrode, respectively. The optical property of compact TiO_2_ (c‐TiO_2_) and ALD SnO_2_ films was compared by measuring transmittance. As shown in Figure 1b, slightly improved transmittance than the FTO substrate from 20 nm thick ALD SnO_2_‐coated film is measured while decreased transmittance is observed from the 20 nm thick TiO_2_‐coated FTO substrate. The ALD SnO_2_ ETL gave a better device performance than the c‐TiO_2_ ETL as shown in the *J–V* curves of Figure 1c. As the low‐temperature‐processed SnO_2_ film is likely to have a similar conduction band position, it is reasonable to find a similar *V*_oc_ with both SnO_2_ and TiO_2_.^13^ The higher short‐circuit current density (*J*_sc_) and FF obtained from the cell with ALD SnO_2_ ETL can be surmised by the better light harvesting and electron extraction at the interface. Despite the negligible difference of light absorption at the longer wavelength as seen in Figure 1d, the better absorption in the shorter wavelength region seems to be beneficial for increasing a current output. As the difference in electron extraction can be mirrored by photoluminescence (PL) emission, we measured PL of perovskite films with FTO/c‐TiO_2_ and FTO/ALD SnO_2_ substrates, respectively. Considering both perovskite layers have the same thickness, charge collection can be differentiated by the interfacial condition. Compared with the TiO_2_/perovskite film, much lower PL intensity, ≈40% reduction, is observed from the SnO_2_/perovskite film as displayed in Figure 1d. A reduced PL intensity with the SnO_2_/perovskite film indicates that the excited electrons in perovskite can be quenched more quickly at the SnO_2_/perovskite interface than that of TiO_2_/perovskite. These results strongly support the reasons of the higher efficiency with the ALD SnO_2_ ETL shown in Figure 1c. As the postannealing at a mild temperature of 180 °C is known to enhance an electrical property of the solution‐processed film, it is necessary to verify if the improvement occurs similarly even with ALD SnO_2_ films.^11^, ^13^ The *J–V* curves of the perovskite cells with postheated films are shown in Figure 1e. For comparison, the SnO_2_ films are deposited at 100 °C, which is slightly lower than reference temperature of 120 °C. An S‐shaped curve with the as‐prepared film maybe owing to incomplete conversion of the precursor which can cause a high series resistance of the device is observed. As expected, highly enhanced FF and a slightly improved *J*_sc_ are displayed after heating the film at 180 °C. However, the film heat‐treated at a higher temperature of 300 °C shows a severe drop of *V*_oc_. The observed phenomenon amazingly equals well with our previous results with SnCl_4_ and water precursors.^13^ In Figure S1 in the Supporting Information, the ALD SnO_2_ films show a dense form without visible morphology change even after postannealing at 300 °C; thus, we can assume that the ALD SnO_2_ film prepared at low temperature also has the passivated tin oxide (PTO) structure.^13^ We also investigated the perovskite film morphology and crystallinity on c‐TiO_2_ and ALD SnO_2_ substrates and found no distinct change by the substrates as seen in Figures S2 and S3 in the Supporting Information, respectively.

**Figure 1:**
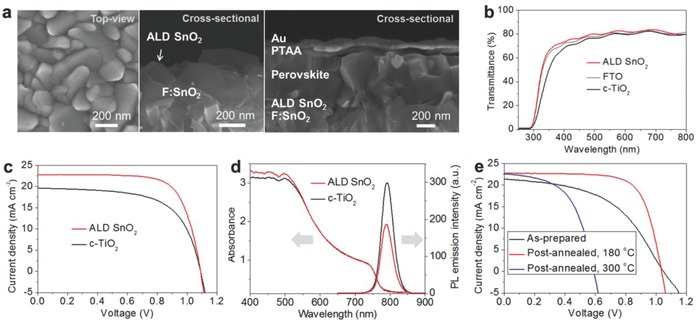
Planar type perovskite solar cells with ALD SnO_2_ ETLs. a) SEM images of the ALD SnO_2_ films and the complete perovskite solar cell. b) Transmittance of the ALD SnO_2_, FTO, and c‐TiO_2_ films. c) *J–V* curves with SnO_2_ and c‐TiO_2_ films. d) Absorbance and photoluminescence emission spectra of the perovskite films formed on the ALD SnO_2_ and c‐TiO_2_ substrates. e) *J–V* curve change according to postannealing of ALD SnO_2_ films.

To see the influence of postannealing temperature, samples deposited at 100 °C on SiO_2_/Si substrate and postannealed at 180 and 300 °C were investigated by tip‐enhanced Raman spectroscopy (TERS) as seen in **Figure** **2**a. One major peak was identified at 469 cm^−1^, which was assigned to the Raman active Eg mode associated with the α_xz_ polarizability tensor,^18^ which indicates the presence of primarily one crystalline orientation in reference to the instrument tip.^19^ To compare the intensities of the SnO_2_ Eg peaks, the TER spectra were normalized to the Si peak at 521 cm^−1^ since the portion of substrate probed is constant. Raman scattering intensity was weakest in the 100 °C deposited sample and strongest in the 300 °C annealed sample, while the 180 °C annealed sample exhibited more intense Raman scattering than the 100 °C sample. This suggests that the films postannealed at higher temperature are more crystalline and of primarily one orientation than the 100 °C annealed film. As the ALD SnO_2_ films prepared at low temperature are known to have an amorphous nature, the result of TERS seems to match well with that of X‐ray diffraction (XRD) spectroscopy.^20^, ^21^ X‐ray photoemission spectroscopy (XPS) analysis was performed to characterize the film property. Strong peaks at 487 and 495 eV attributed to Sn^4+^ ions indicate the formation of SnO_2_ and small shift according to annealing temperature, which implies that the chemical change of the film is seen in Figure 2b.^22^ The higher intensities in the postannealed films reveal that the crystallinity of ALD SnO_2_ film has been improved, which is consistent with obtained values from TER analysis. A weak asymmetry shown in all spectra can be reasoned by Sn^2+^ ions originated from oxygen vacancy (*V*_o_) of the SnO_2_ surface, which is assumed as the origin of the high conductivity of the SnO_2_ film.^12^, ^22^ Strong asymmetry of the spectra indicating the presence of subbonds is also observed in O s1 region (Figure 2c), which can be deconvoluted into two subpeaks as shown in Figure S4 in the Supporting Information. The fitted peak with higher intensity at 530.2 eV should be assigned to the binding energy for O—S bond (Sn^4+^), while the small one at 531.5 eV can be associated with oxygen impurities such as OH ligands and water adsorption on the film.^20^, ^21^, ^22^ The impurity peak is still found even after postannealing at 300 °C with considerable intensity. In contrast, we can find a distinct change of N 1s intensity at 400 eV, which is attributed to N—Sn and N—C bonds as displayed in Figure 2d. It is seen that the highest intensity of the as‐prepared film slightly decreases after 180 °C annealing, while no visible N bond appears after annealing at 300 °C. The result indicates that the unreacted precursor remains on the films prepared at the low‐temperature deposition (as‐prepared film) and annealed at 180 °C, as discussed in the previous reports.^20^, ^23^ The expected reaction during the ALD‐based process is illustrated in Figure 2e. For the complete conversion, the Sn—N bond should be broken and reacted by oxygen to form SnO_2_, otherwise, the residual tetrakis‐dimethyl‐amine‐tin (TDMASn) remains as an impurity on the film.^20^, ^23^ To better understand, we analyzed the TDMASn precursor by thermogravimetry (TG) and differential scanning calorimetry (DSC) as shown in Figure 2f. It is seen that the weight loss occurs rapidly in a range between 70 and 150 °C, with a total weight loss of ≈92%. The DSC result shows one endothermic peak at 230 °C, indicating that the decomposition of the precursor and the residual precursor can coexist with SnO_2_ below the temperature.^23^, ^24^ From our observation, we can draw a probable structure of the SnO_2_ ETL as depicted in Figure 2g. The structure contains highly conductive crystalline SnO_2_ self‐passivated by the properly controlled residual precursor, which provides good electron mobility and hole‐blocking ability as well as an amorphous nature of the PTO layer. When combining with device results shown in Figure 1e, it becomes clearer that the passivation of SnO_2_ is the key for the high efficiency.^13^

**Figure 2:**
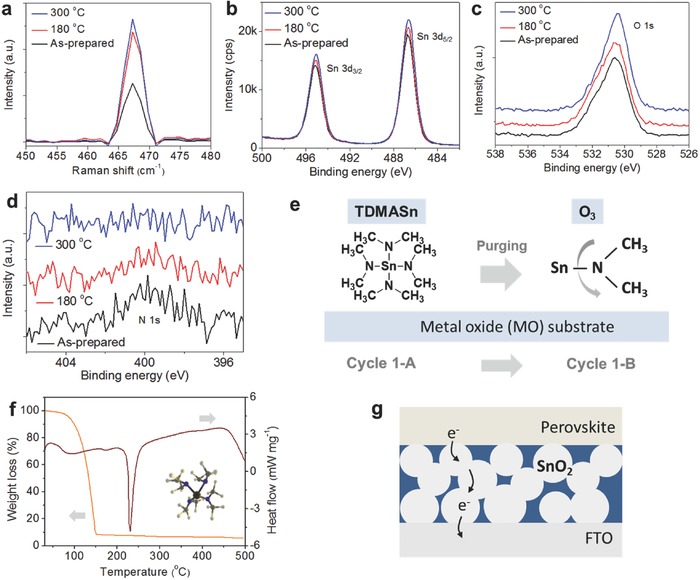
Characterization of the surface property of the ALD SnO_2_ film as a function of postannealing temperature. a) TERS result. b–d) XPS spectra. e) Schematic of the probable reaction during ALD deposition using TDMASn and O_3_ precursor. f) TG and DSC spectra of the TDMASn precursor. g) The proposed structure of the low‐temperature‐processed ALD SnO_2_ film. The SnO_2_ particles are self‐passivated by the residual precursor depicted as a blue region.

The energy band structure of the SnO_2_ films is displayed in **Figure** **3**. Figure 3a,b shows the secondary electron cut‐off binding energy and the valence band (VB) edges of SnO_2_ films measured by ultraviolet photoelectron spectroscopy (UPS), respectively. The exact VB offset was determined by extrapolating the leading edge of the VB spectrum to the base line. From the spectra, VB offsets of as‐deposited, 180, and 300 °C films are measured as 3.61, 3.51, and 3.30 eV, respectively. We measured bandgaps by reflection electron energy loss spectroscopy (REELS).^25^ The bandgaps of SnO_2_ thin films are determined by the difference between elastic peak and onset of inelastic loss spectrum. Hence, we measured bandgap energies using an intercept of the line with a maximum negative slope near the edge to the background level as shown in Figure 3c. The bandgap energies of as‐deposited, 180, and 300 °C films are measured as 4.38, 4.33, and 3.93 eV, respectively. This result shows that the bandgaps decreased as the annealing temperature increased. Fermi level was calculated using the equation, *E*_F_ = *E*_cut‐off_ – 40.8 eV (irradiation energy of He II source). Thus, Fermi levels for the films can be calculated as −4.16, −4.32, and −4.51, respectively. And the valence band maximum (VBM) was determined by the equation, VBM = *E*_F_ – VB offset. It should be noted that three different films have similar VBMs of −7.77, −7.83, and −7.81 eV, while the gradual downward shift of *E*_F_ and conduction band maximum (CBM) of annealed films are observed in Figure 3d as similarly seen in the recent report.^26^ The summary of the values from the measurement is displayed in Table S1 in the Supporting Information. In Figure 3e, we depicted aligned energy levels of the SnO_2_ films under heterojunction with FTO and perovskite. In the dark condition, Fermi levels of SnO_2_ films and perovskite (n‐type) are pinned by FTO.^27^ It is interesting that all samples show similar values of CB offsets of 0.76, 0.79, and 0.75 after alignment, which indicates that the electron injection from perovskite is hardly influenced by the downward shift of CBM and *E*_F_ of SnO_2_ films. Thus, the result is partially opposite to the general understanding that the lower position of the conduction band is beneficial for electron injection. Instead, we can conclude that the improved efficiency observed from the cells with the annealed SnO_2_ films at low temperature is dominantly by the reduced series resistance of the SnO_2_ layer with properly controlled surface passivation.

**Figure 3:**
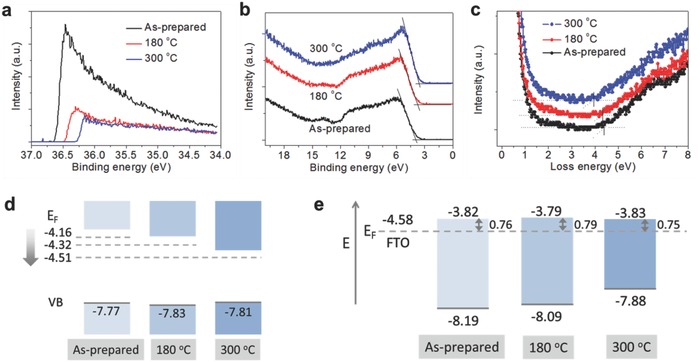
Energy band structure of the ALD SnO_2_ films according to postannealing temperature. a) Cut‐off binding energy measured by UPS. b) Valence band edge measured by UPS. c) Bandgap of the SnO_2_ films measured by REELS. d) Bandgap edges and Fermi levels of the SnO_2_ films as a function of annealing temperature. e) Estimated CB offsets after Fermi level alignment under heterojunction with FTO and perovskite (in the dark).

So far, much of research interest related to ALD SnO_2_ was placed on enhancing electrical conductivity with high transparency for the conductive film applications. Accordingly, the residual precursor was regarded as rather harmful by increasing resistivity of the film. However, our findings demonstrate that such impurities can be beneficial or essential for photovoltaics by passivating surface defects of the SnO_2_ layer if it is controlled properly. Passivation of SnO_2_ can be easily controllable by manipulating the deposition and postannealing temperature as illustrated in **Figure** **4**. We mainly examined the influence of postannealing temperature for ALD SnO_2_ films, but the result with different deposition temperature displays a remarkably analogous trend as shown in Figure S5 in the Supporting Information. A strong recombination pathway that may occur when the SnO_2_ film lacks hole‐blocking ability is depicted in Figure S6 in the Supporting Information. For comparison, we fabricated planar type cells with a thin layer of SnO_2_ nanoparticles,^28^ and found a strong shunt as seen in Figure S7 in the Supporting Information. From this insight, we can draw strategies for practical passivation of the SnO_2_ layer, which is classified as 4 types as sketched in Figure 4. The illustration suggests that postpassivation denoted as types 2, 3, and 4 also can be a good option to passivate the SnO_2_ layer.

**Figure 4:**
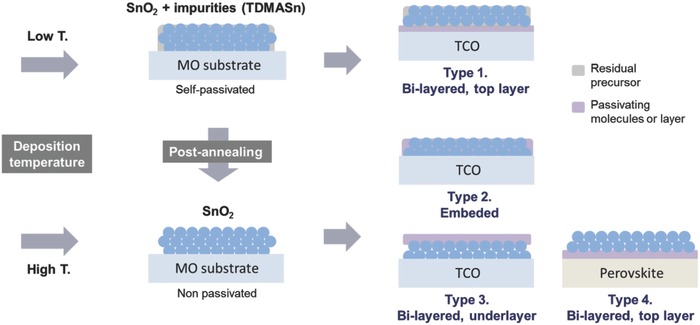
Self‐ and postpassivation of the ALD SnO_2_ films. A low‐temperature deposition results in incomplete conversion of the precursor, which remains on the SnO_2_ film as a self‐passivating layer. The schematic shows that the surface passivation of SnO_2_ films can be controlled by postannealing. Four different methods of postpassivation are proposed.

In this work, we introduced “Type 1” passivation, which combines a thin TiO_2_ underlayer with a PTO top layer,^13^ and achieved enhanced PCEs as seen in Table S2 in the Supporting Information. A *J*−*V* curve for the champion cell is shown in **Figure** **5**a. The best device shows excellent photovoltaic values in a planar structure with the *J*_sc_ of 22.67 mA cm^−2^, the *V*_oc_ of 1.13 V, the FF of 0.78, and the PCE of 20.03% under 1 sunlight illumination condition when scanned backward. In the external quantum efficiency (EQE) measurement, we confirmed that the *J*_sc_ from the solar simulator (1 sun, Xe lamp) agrees with the integrated *J*_sc_ value of 23.03 mA cm^−2^ as seen in Figure 5b. In Figure S8 in the Supporting Information, a small *J–V* curve hysteresis is still observed as similarly reported in our previous paper.^13^ For the long‐term stability test, cells were stored in the dark condition without encapsulation (in the air, relative humidity, ≈20%) after every measurement. It is known that the better interfacial condition between SnO_2_ and perovskite compared with that of TiO_2_/perovskite can also result in better stability of the perovskite layer.^29^ A promising result was observed that the PTO‐based perovskite cells have an excellent stability over 90 days, while a cell with the c‐TiO_2_ ETL degrades quickly as shown in Figure 5c.

**Figure 5:**
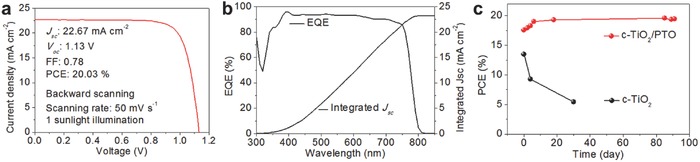
Performance of the best device with the bi‐layered ETL. a) *J–V* curve of the champion cell. b) EQE and integrated *J*_sc_. c) Long‐term stability of the device without encapsulation.

In summary, we investigated low‐temperature‐processed ALD SnO_2_ films for perovskite solar cells and found that the SnO_2_ ETL should be passivated due to metal‐like nature of SnO_2_. We unveil that the residual precursor, TDMASn, on the ALD SnO_2_ film can be a good self‐passivating material. It was found that chemical and electrical properties of the ALD SnO_2_ film were strongly associated with deposition and postannealing temperature. By investigating optical, chemical, and electrical properties of the ALD SnO_2_ films, we found that the charge collection from perovskite to SnO_2_ can be less influenced by the downward shift of CBM and *E*_F_ of SnO_2_ films, but strongly affected by crystallinity and proper surface passivation of the SnO_2_ layer. Additionally, the bi‐layered ETL of c‐TiO_2_/passivated SnO_2_ was confirmed to provide better hole‐blocking ability than a single‐passivated SnO_2_ layer, which resulted in the further enhanced PCE. Our findings highlight the importance of surface passivation for SnO_2_‐based ETLs, and explain why the low‐temperature process is necessary to obtain high PCEs. We suggest the possibility of better PCEs by efficient passivation with diverse materials for high‐efficiency solar cells.

## Experimental Section

*Device Fabrication*: FTO glass (Nippon Sheet Glass) substrates were partially etched with Zn powder and diluted HCl solution, and sequentially cleaned with detergent solution, water, and ethanol. ALD SnO_2_ films were prepared with tetrakis‐dimethyl‐amine tin as a Sn precursor and ozone as an oxygen reactant.^22^ The deposition of ALD SnO_2_ was carried out at 120 or 100 °C, and the films were used as‐prepared or after postannealing. For Type 1 passivation, a compact TiO_2_ layer was coated on the cleaned FTO substrate by spray pyrolysis deposition at 450 °C with a precursor solution prepared by diluting titanium diisopropoxide (Sigma‐Aldrich) in ethanol. The PTO layer was formed on a c‐TiO_2_‐coated FTO films by ALD. The deposition was carried out at 100 °C, and the as‐prepared film was postannealed at 180 °C for 1 h. A thin layer of SnO_2_ nanoparticles was prepared by spin coating the SnO_2_ colloid precursor (Alfa Aesar, ≈2.7%). The SnO_2_ layer was spin coated at 4000 rpm for 30 s, and heat‐treated in the air at 150 °C for 30 min.^28^ The (FAPbI_3_)_0.85_(MAPbBr_3_)_0.15_ precursor solution was prepared by mixing PbI_2_ (1.10 m, TCI), FAI (1.05 m, Dyesol), PbBr_2_ (0.185 m, TCI), and MABr (0.185 m, Dyesol) in a mixed solvent of DMF:DMSO = 4:1 (volume ratio). The solution was spin coated at 1000 rpm for 10 s and, continuously at 5000 rpm for 30 s. During the second step, 100 µL of chlorobenzene was poured on the film at 15 s. Films are postannealed at 100 °C for 60 min. The HTM solution was prepared by dissolving 10 mg of PTAA (Emindex) with additives in 1 mL of toluene. As additives, 7.5 µL of Li‐bis(trifluoromethanesulphonyl) imide (Aldrich) from the stock solution (170 mg in 1 mL of acetonitrile), and 4 µL of 4‐*tert*‐butylpyridine were added. The HTM layer was formed by spin coating the solution at 3000 rpm for 30 s, and followed by the deposition of the 70 nm thick Au electrode by a thermal evaporation.

## Conflict of Interest

The authors declare no conflict of interest.

## Supporting information

Supplementary
