# Supplementary material for: Efficient Planar Perovskite Solar Cells Using Passivated Tin Oxide as an Electron Transport Layer
Source: Adv Sci (Weinh). 2018 Mar 25;5(6):1800130. doi: 10.1002/advs.201800130 (PMC6010698; doi:10.1002/advs.201800130)
Supplement: Supplementary file 1 — Supplementary [file ADVS-5-1800130-s001.pdf]

Copyright WILEY-VCH Verlag GmbH & Co. KGaA, 69469 Weinheim, Germany, 2018.

ADVANCED SCIENCE
Open Access

Supporting Information

for *Adv. Sci.,* DOI: 10.1002/advs.201800130

Efficient Planar Perovskite Solar Cells Using Passivated Tin Oxide as an Electron Transport Layer

Yonghui Lee, Seunghwan Lee, Gabseok Seo, Sanghyun Paek, Kyung Taek Cho, Aron J. Huckaba, Marco Calizzi, Dong-won Choi, Jin-Seong Park, Dongwook Lee, Hyo Joong Lee, Abdullah M. Asiri, and Mohammad Khaja Nazeeruddin**

Copyright WILEY-VCH Verlag GmbH & Co. KGaA, 69469 Weinheim, Germany, 2016.

Supporting Information

Efficient planar perovskite solar cells using passivated tin oxide as an electron transport layer

Yonghui Lee, Seunghwan Lee, Gabseok Seo, Sanghyun Paek, Kyung Taek Cho, Aron J. Huckaba, Marco Calizzi, Dong-won Choi, Jin-Seong Park, Dongwook Lee, Hyo Joong Lee, Abdullah M. Asiri, and Mohammad Khaja Nazeeruddin**

Film and Device Characterization

Scanning electron microscopy (SEM) measurements were carried out on a high-resolution ZEISS Merlin at 5 kV, and images were acquired with the in-lens detector.

Absorbance and reflectance were measured with an integrating sphere using a UV/Vis/NIR spectroscopy (PerkinElmer Lambda 950S). Photoluminescence emission was measured with a Fluorescence spectrometer (PerkinElmer LS 55).

Tip-Enhanced Raman Spectroscopy (TERS) measurements were performed using a Renishaw InVia Raman spectrometer coupled to a Nanonics MultiView 2000 Atomic Force Microscope (AFM). A 532 nm CW laser was used as excitation source, focused on the AFM tip apex from the top through an external Olympus microscope using a super-long working distance objective (Nikon 50×, 0.40 NA, 22 mm WD). The Raman signal was dispersed by a grating with 2400 grooves mm^{-1} and collected by a CCD detector. The AFM tip was commercial (Nanonics), coated with Au nanoparticles with diameter between 100 nm and 200 nm. The tip was precisely aligned under the laser by maximizing the intensity ratio of the signal coming from the SnO_2 thin film and the Si substrate.

Thermogravimetry (TG) and differential scanning calorimetry (DSC) were done with a NETZSCH 200-F3.

XPS analysis was done by an equipment (Quantum 2000 system) with Al kα x-ray source (*hv*: 1486.6 eV) and a hemispherical electron analyzer. The XPS results provide the average chemical information on spatial area of 100 μm diameter and depth of 5~10 nm.

UPS measurements were performed with a home-made photoemission spectroscopy system, which consists of the ultraviolet source (VUV 5000) and an electron analyzer in ultrahigh vacuum (UHV) chamber. The He II source of 40.8 eV, controlled using monochromator, enabled to obtain precise electronic structures from UPS spectra.

REELS was measured by a Auger electron spectroscopy (AES; VG Microlab 310 system) with a concentric hemispherical analyzer (CHA). The primary energy of electrons for the AES measurement was 1.5 keV and the full width at half maximum (FWHM) of the elastic peaks of 1.5 keV was 0.8 eV.

Solar cell measurement was done using an Oriel solar simulator (450W Xenon, AAA class). The light intensity was calibrated with a Si reference cell equipped with an IR-cutoff filter (KG3, Newport), and it was recorded prior to measurement. Current-voltage characteristics of the cells were obtained by applying an external voltage bias while measuring the current response with a digital source meter (Keithley 2400). The voltage scan rate was 50 mV s^{-1} and no device preconditioning such as light soaking or forward voltage bias applied for long time, was applied before starting the measurement. The cells were masked with the active area of 0.16 cm^2 to fix the active area and reduce the influence of the scattered light. EQE was measured with IQE200B (Oriel) without bias light.

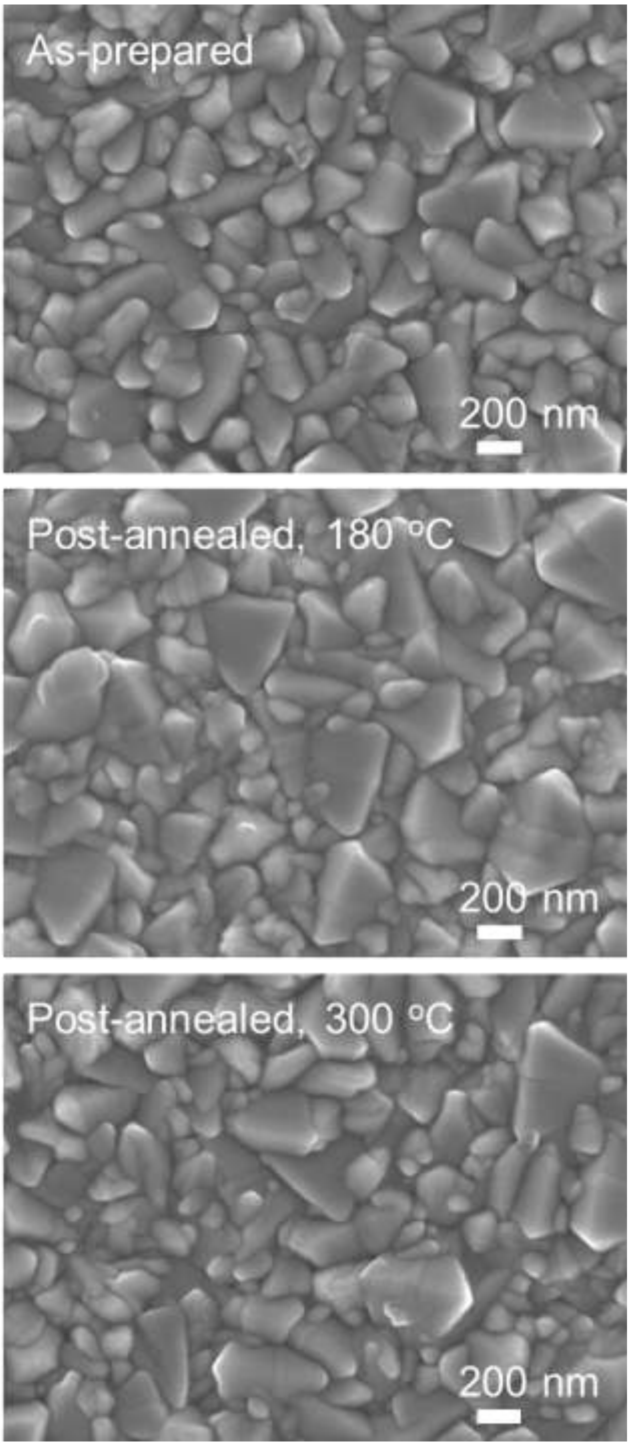

Figure S1. SEM top-view images of the ALD SnO_2 films according to post-annealing temperature.

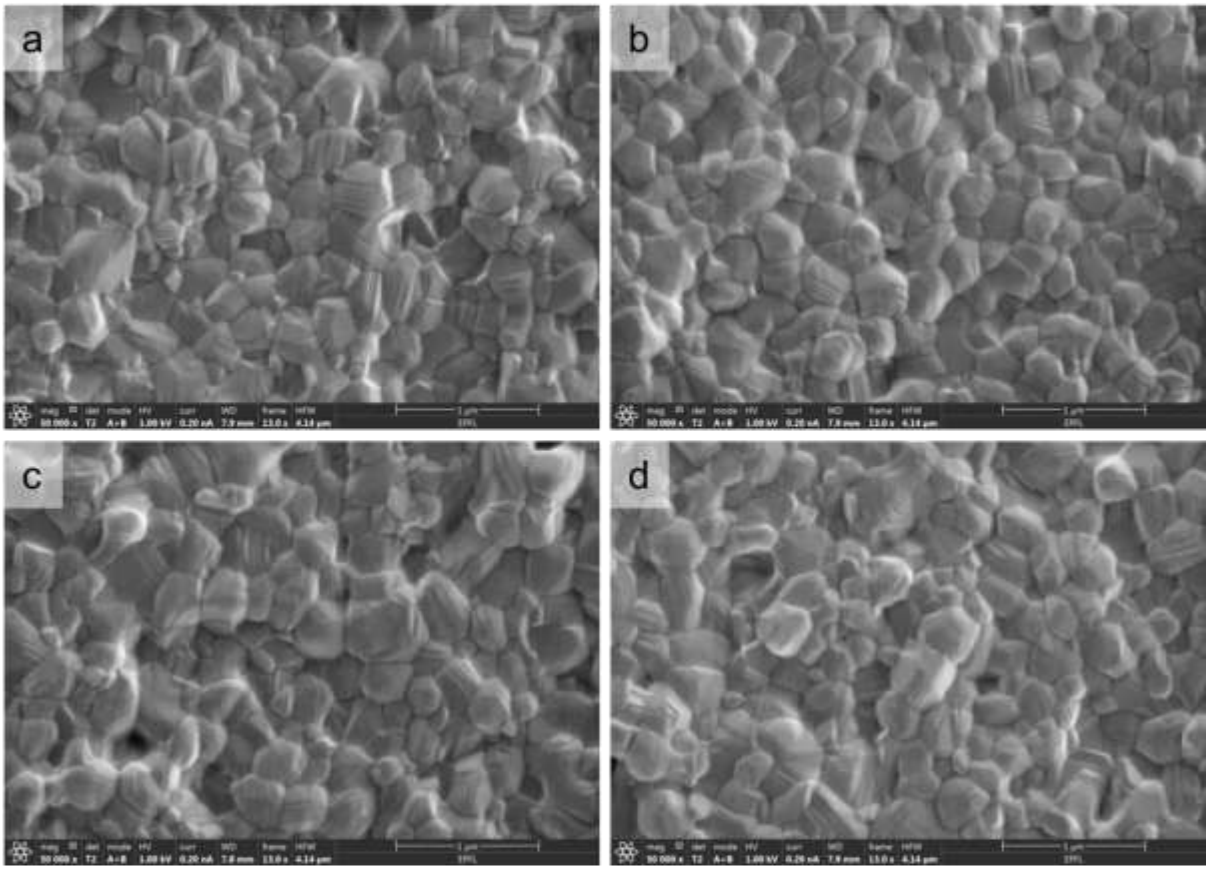

Figure S2. SEM top-view images of the perovskite films formed on a) c-TiO_2, b) ALD SnO_2 as-prepared, c) ALD SnO_2 post-annealed at 180 °C, and d) ALD SnO_2 post-annealed at 300 °C substrates, respectively.

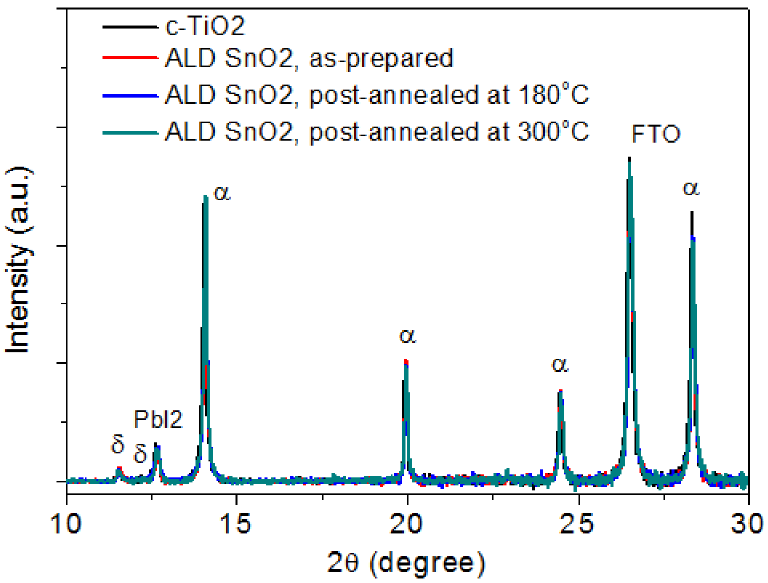

Figure S3. X-ray diffraction (XRD) patterns of the $(FAPbI_3)_{0.85}(MAPbBr_3)_{0.15}$ perovskite films formed on c-TiO_2, ALD SnO_2 (as-prepared, post-annealed at 180 °C and 300 °C) substrates.

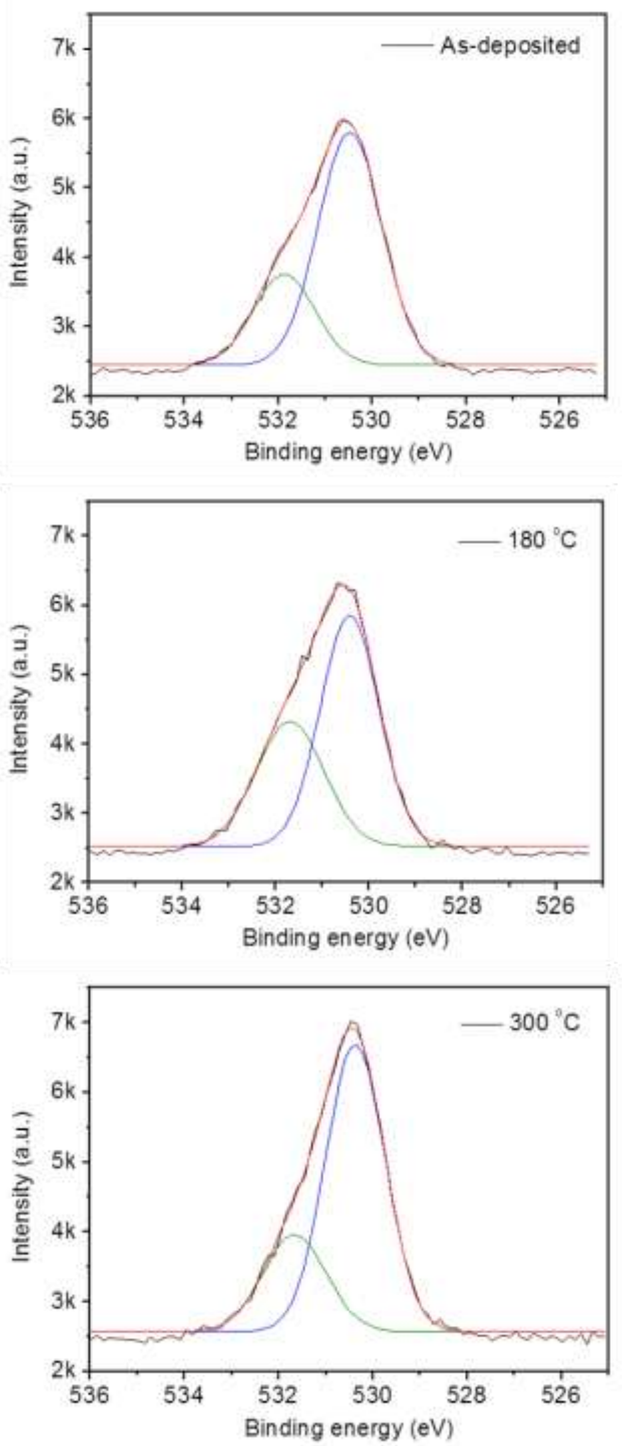

Figure S4. Fitting of the XPS O s1 core level spectra.

Table S1. Summary of the values measured by UPS and REELS.

Samples	Secondary-cutoff	Work function	Valence band offset	Ionization potential [VBM]	Energy bandgap	Conduction band [CBM]
As-prepared	36.64	4.16	3.61	-7.77	4.38	-3.4
180 °C	36.48	4.32	3.51	-7.83	4.33	-3.53
300 °C	36.29	4.51	3.30	-7.81	3.93	-3.76

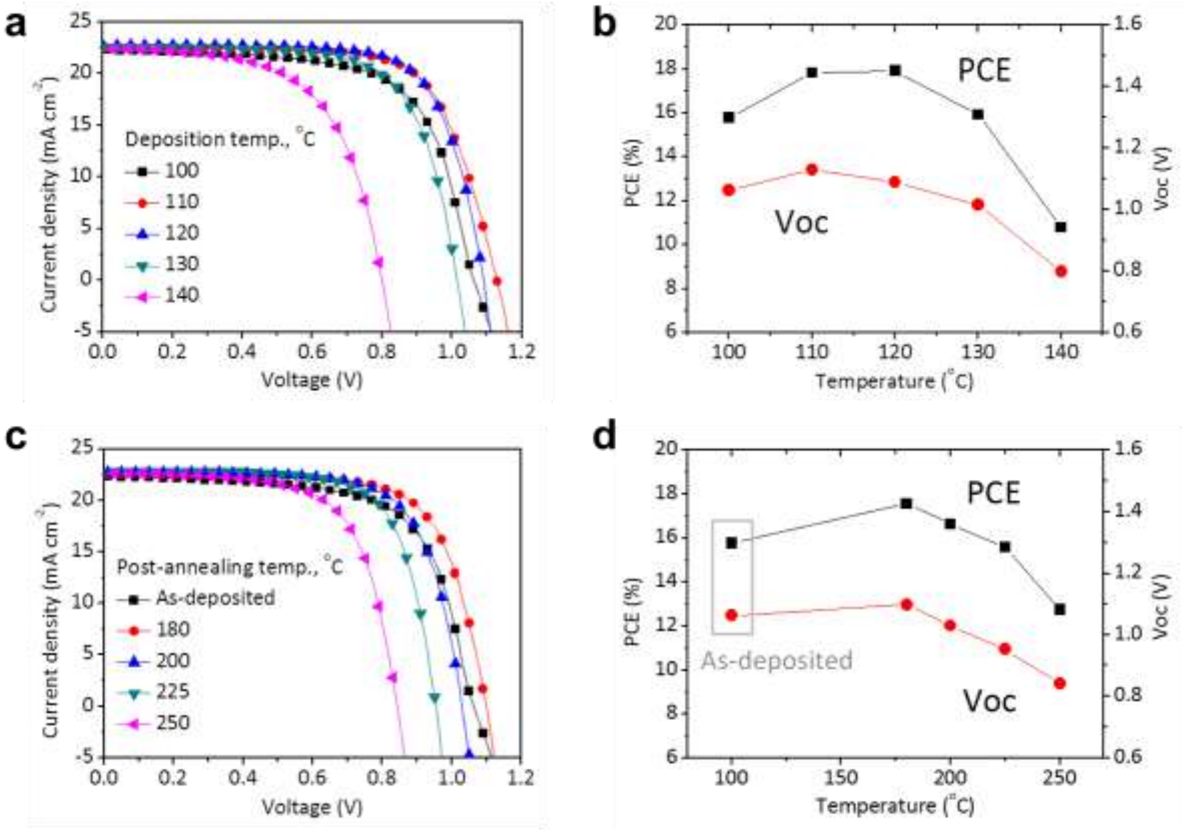

Figure S5. Influence of deposition and post-annealing temperature of SnO_2 films on device performance. a, *J*-*V* curves according to deposition temperature. b, PCE and V_{oc} change of (a). c. *J*-*V* curves according to post-annealing temperature. d, PCE and V_{oc} change of (c).

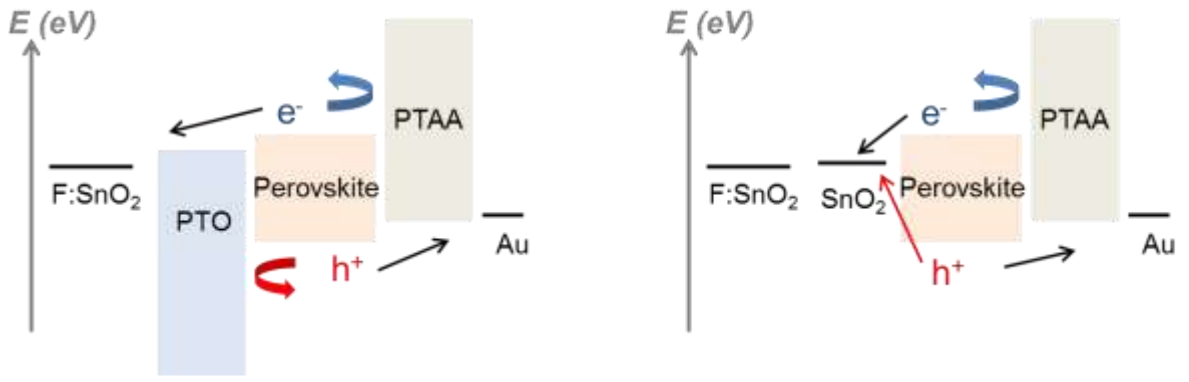

Figure S6. Comparison of hole-blocking ability between passivated SnO_2 and non-passivated SnO_2 ETLs. Due to metal-like nature of non-passivated SnO_2, a strong recombination is expected at the non-passivated SnO_2/perovskite interface.

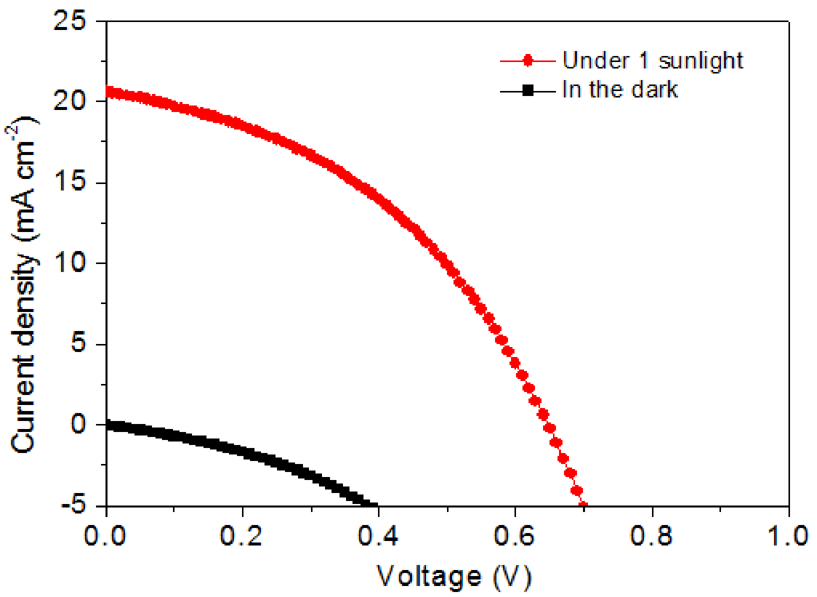

Figure S7. *J-V* curve of the perovskite solar cell with a thin layer of SnO_2 nanoparticles.

Table S2. *J-V* curve values of the cells with PTO and TiO_2/PTO ETLs. The average values are obtained from 5 cells in a batch. Solar cells are scanned backward under 1 sunlight illumination.

ETL	J_{sc} [mA cm^{-2}]	V_{oc} [V]	FF	PCE [%]
PTO	22.59 ± 0.15	1.07 ± 0.04	0.73 ± 0.03	17.75 ± 0.62
TiO_2/PTO	22.68 ± 0.30	1.13 ± 0.01	0.78 ± 0.01	19.83 ± 0.20

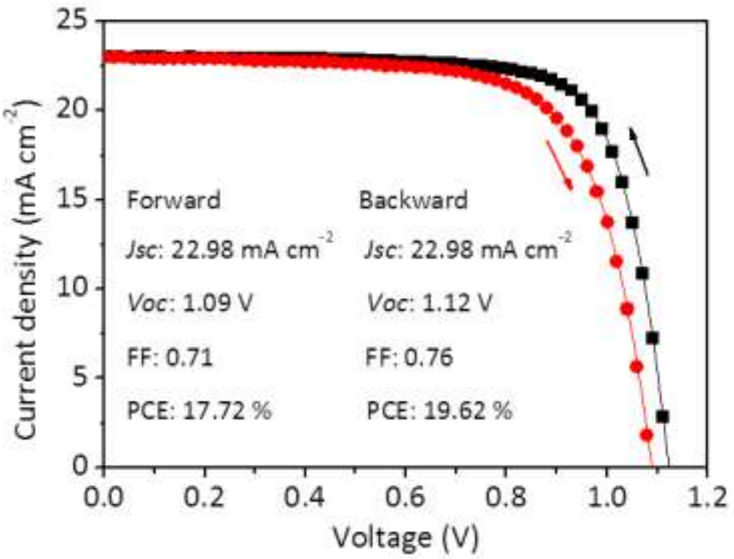

Figure S8. The *J*-*V* curve hysteresis according to the scanning direction.
